# Hypothesis-generating and confirmatory studies, Bonferroni correction, and pre-specification of trial endpoints

**DOI:** 10.1080/17453674.2019.1612624

**Published:** 2019-05-14

**Authors:** Jonas Ranstam

A p-value presents the outcome of a statistically tested null hypothesis. It indicates how incompatible observed data are with a statistical model defined by a null hypothesis. This hypothesis can, for example, be that 2 parameters have identical values, or that they differ by a specified amount. A low p-value shows that it is unlikely (a high p-value that it is not unlikely) that the observed data are consistent with the null hypothesis. Many null hypotheses are tested in order to generate study hypotheses for further research, others to confirm an already established study hypothesis. The difference between generating and confirming a hypothesis is crucial for the interpretation of the results. Presenting an outcome from a hypothesis-generating study as if it had been produced in a confirmatory study is misleading and represents methodological ignorance or scientific misconduct.

Hypothesis-generating studies differ methodologically from confirmatory studies. A generated hypothesis must be confirmed in a new study. An experiment is usually required for confirmation as an observational study cannot provide unequivocal results. For example, selection and confounding bias can be prevented by randomization and blinding in a clinical trial, but not in an observational study. Confirmatory studies, but not hypothesis-generating studies, also require control of the inflation in the false-positive error risk that is caused by testing multiple null hypotheses. The phenomenon is known as a multiplicity or mass-significance effect. A method for correcting the significance level for the multiplicity effect has been devised by the Italian mathematician Carlo Emilio Bonferroni. The correction (Bender and Lange 2001) is often misused in hypothesis-generating studies, often ignored when designing confirmatory studies (which results in underpowered studies), and often inadequately used in laboratory studies, for example when an investigator corrects the significance level for comparing 3 experimental groups by lowering it to 0.05/3 = 0. 017 and believes that this solves the problem of testing 50 null hypotheses, which would have required a corrected significance level of 0.05/50 = 0.001.

In a confirmatory study, it is mandatory to show that the tested hypothesis has been pre-specified. A study protocol or statistical analysis plan should therefore be enclosed with the study report when submitted to a scientific journal for publication. Since 2005 the ICMJE (International Committee of Medical Journal Editors) and the WHO also require registration of clinical trials and their endpoints in a publicly accessible register before enrollment of the first participant. Changing endpoints in a randomized trial after its initiation can in some cases be acceptable, but this is never a trivial problem (Evans 2007) and must always be described to the reader. Many authors do not understand the importance of pre-specification and desist from registering their trial, use vague or ambiguous endpoint definitions, redefine the primary endpoint during the analysis, switch primary and secondary outcomes, or present completely new endpoints without mentioning this to the reader. Such publications are simply not credible, but are nevertheless surprisingly common (Ramagopalan et al. 2014) even in high impact factor journals (Goldacre et al. 2019). A serious editorial evaluation of manuscripts presenting confirmatory results should always include a verification of the endpoint’s pre-specification.

Hypothesis-generating studies are much more common than confirmatory, because the latter are logistically more complex, more laborious, more time-consuming, more expensive, and require more methodological expertise. However, the result of a hypothesis-generating study is just a hypothesis. A hypothesis cannot be generated and confirmed in the same study, and it cannot be confirmed with a new hypothesis-generating study. Confirmatory studies are essential for scientific progress.

**Jonas Ranstam**, Statistical Editor
jonas.ranstam@gmail.com


## References

[CIT0001] Bender R, Lange S Adjusting for multiple testing: when and how? J Clin Epidemiol 2001; 54: 343–9. 11297884 10.1016/s0895-4356(00)00314-0

[CIT0002] Evans S When and how can endpoints be changed after initiation of a randomized clinical trial? PLoS Clin Trials 2007; 2: e18. 17443237 10.1371/journal.pctr.0020018 PMC1852589

[CIT0003] Goldacre B, Drysdale H, Milosevic I, Slade E, Hartley P, Marston C, Powell-Smith A, Heneghan C, Mahtani K R COMPare: a prospective cohort study correcting and monitoring 58 misreported trials in real time. Trials 2019; 20: 118. 30760329 10.1186/s13063-019-3173-2 PMC6375128

[CIT0004] Ramagopalan S, Skingsley A P, Handunnetthi L, Klingel M, Magnus D, Pakpoor J, Goldacre B Prevalence of primary outcome changes in clinical trials registered on ClinicalTrials.gov: a cross-sectional study. F1000Research 2014, 3: 77. 25075294 10.12688/f1000research.3784.1 PMC4032105

